# Wind Farm Facilities in Germany Kill Noctule Bats from Near and Far

**DOI:** 10.1371/journal.pone.0103106

**Published:** 2014-08-13

**Authors:** Linn S. Lehnert, Stephanie Kramer-Schadt, Sophia Schönborn, Oliver Lindecke, Ivo Niermann, Christian C. Voigt

**Affiliations:** 1 Department of Evolutionary Ecology, Leibniz Institute for Zoo and Wildlife Research, Berlin, Germany; 2 Central Repository for Natural Science Collections, Martin-Luther-University Halle-Wittenberg, Halle (Saale), Germany; 3 Institute of Environmental Planning, Leibniz Universität Hannover, Hannover, Germany; University of Southern Denmark, Denmark

## Abstract

Over recent years, it became widely accepted that alternative, renewable energy may come at some risk for wildlife, for example, when wind turbines cause large numbers of bat fatalities. To better assess likely populations effects of wind turbine related wildlife fatalities, we studied the geographical origin of the most common bat species found dead below German wind turbines, the noctule bat (*Nyctalus noctula*). We measured stable isotope ratios of non-exchangeable hydrogen in fur keratin to separate migrants from local individuals, used a linear mixed-effects model to identify temporal, spatial and biological factors explaining the variance in measured stable isotope ratios and determined the geographical breeding provenance of killed migrants using isoscape origin models. We found that 72% of noctule bat casualties (n = 136) were of local origin, while 28% were long-distance migrants. These findings highlight that bat fatalities at German wind turbines may affect both local and distant populations. Our results indicated a sex and age-specific vulnerability of bats towards lethal accidents at turbines, i.e. a relatively high proportion of killed females were recorded among migratory individuals, whereas more juveniles than adults were recorded among killed bats of local origin. Migratory noctule bats were found to originate from distant populations in the Northeastern parts of Europe. The large catchment areas of German wind turbines and high vulnerability of female and juvenile noctule bats call for immediate action to reduce the negative cross-boundary effects of bat fatalities at wind turbines on local and distant populations. Further, our study highlights the importance of implementing effective mitigation measures and developing species and scale-specific conservation approaches on both national and international levels to protect source populations of bats. The efficacy of local compensatory measures appears doubtful, at least for migrant noctule bats, considering the large geographical catchment areas of German wind turbines for this species.

## Introduction

Over the past two decades, wind energy has continuously been promoted in many European and North American countries and is currently widely recognized as an environmentally friendly alternative to conventional energy production such as those based on nuclear and fossil fuels. However, numerous studies indicate that the establishment of wind turbines may involve negative effects on wildlife species, reaching from direct impacts such as lethal collisions of birds and bats with turbine structures [1], [2], [3] to indirect threats like habitat fragmentation [4]. Recent bat fatality surveys revealed that the numbers of bats killed annually at European and North American wind turbines is alarming [2], [5], [6], with estimates of hundreds of thousands of bats killed by wind turbines in the U.S.A. [7]. On the European continent, Germany and neighboring countries appear to be of particular geographical importance for the connectivity between breeding and wintering areas of many long distance migrants from north-eastern bat populations [8], [9]. Considering that the density of onshore wind turbines is among the highest worldwide in Germany [10] and is projected to expand further in the future [11], immediate action seems advisable to prevent large-scale losses of wildlife individuals and expected declines in wildlife populations.

Some European bat species undertake long-distance migrations between their breeding habitats in Northeastern Europe and wintering sites in Central or Southern Europe, covering one-way distances of up to 1,900 km [8], [9], [12]. The majority of bat fatalities in Europe occurs during the migration period [5], [6], and is most often affecting species with documented migratory behaviour such as *Nyctalus noctula, Pipistrellus nathusii, N. leisleri* and *Vespertilio murinus* (Dürr 2009 cited in [5]). However, it is unknown whether killed bats are of local origin or originate from distant populations. In some bat species, migratory strategies vary geographically [13], [14], i.e. it is possible to encounter at the same place and at the same time during the migratory period sedentary individuals from local breeding populations as well as migratory individuals of the same species. Accordingly, it is difficult to assess, if bat carcasses found below wind turbines are of local or distant origin. Yet, information on the migratory status of killed bats is a crucial prerequisite to determine their breeding origin and, consequently, to assess the geographical catchment area of bat fatalities at wind turbines. Furthermore, this information would help to assess if fatalities at wind turbines are associated with migratory behaviour. Currently, all European bat species are listed as endangered and protected under the ‘EU Habitats Directive 92/43/CEE (Annexes II and IV)’. Moreover, migratory species, including bats, are covered by the ‘Convention of Migratory Species of Wild Animals’ (Bonn 1979) through the EUROBATS agreement (London 1991), thereby benefiting from legal protection. However, bat species are not protected under the same legal framework in all countries of potential origin, because e.g. non-E.U. countries are not signers of the ‘Habitats Directive’. Considering the presumed large numbers of killed bats and the low capacity of bat populations for recovering from increased mortality rates [15], an improved understanding of the location of affected source populations is essential to evaluate the impact of wind turbines on breeding populations and to formulate and evaluate effective conservation measures.

In this study, we therefore estimated the breeding origin of *Nyctalus noctula* killed at wind turbines in eastern Germany. This aerial-hawking insectivorous bat may cover distances of up to 1,600 km during its annual migrations [16] and individuals are found dead below German wind turbines more often than any other bat species (Dürr 2009 cited in [5]). Here, we used a stable isotope approach to determine the percentage of local and migratory noctule bats killed at wind turbines and to assess the breeding provenance of migrating individuals.

Information on large-scale isotopic patterns in meteoric waters across continents is frequently used for tracking migratory movements of animals [13], [17], [18], [19] because stable isotope ratios of non-exchangeable hydrogen in inert keratin tissues such as fur reflect the variation of stable isotope ratios in precipitation assimilated along the food chain [20], [21]. In Europe, stable isotope ratios of precipitation water are known to vary along a north to south gradient following gradients of annual ambient temperature and rainfall [22] (Bowen et al. 2005). Since noctule bats usually moult in their breeding habitat before the onset of migration [23], [24], [25], they carry an isotopic ‘fingerprint’ of their geographical breeding origin incorporated in their fur [17]. Low values of non-exchangeable hydrogen in fur keratin (δ^2^H_f_) indicate that animals have moulted in a relatively cold climate where δ^2^H precipitation values are low, whereas δ^2^H_f_ are high when bats originate from an area with warm climate where δ^2^H of precipitation is high. Accordingly, it is possible to then distinguish between local and migrant individuals at a given observation site.

We hypothesized that noctule bats (*Nyctalus noctula*) found dead below wind turbines in the eastern part of Germany stem from large catchment areas and are composed of local as well as migratory individuals from distant populations [19], because some individuals of this species conduct long-distance migrations of up to 1,600 km. We expected to detect differences in stable isotope ratios in the non-exchangeable hydrogen of fur keratin (δ^2^H_f_) of carcasses originating from local and distant source populations. Furthermore, we predicted that information on migratory behaviour and seasonality are most important for explaining the variability in δ^2^H_f_ of noctule bat fatalities. In a first step, we categorized individual noctule bats as local or migratory by comparing the measured δ^2^H_f_ of bat carcasses with those expected for sedentary *N. noctula* of the sampling location using an established regression model [13]. Subsequently, we tested the explanatory power of spatial, temporal and biological variables such as sex, season and migratory status (originating from local or distant populations according to stable isotope ratios; see first step above), in a linear mixed-effects model to assess their importance in explaining the measured δ^2^H_f_. In a third step, we applied spatial isoscape origin models based on δ^2^H_f_ and information on preferred cardinal headings of *N. noctula* during spring migration to project the breeding provenance of migratory individuals. Our study demonstrates a trans-boundary impact of German wind energy facilities on an endangered and protected migratory bat species and highlights the importance of an internationally coordinated conservation management plan for noctule bats and other European bat species.

## Methods

### Sample collection and stable isotope analysis

**Figure 1:**
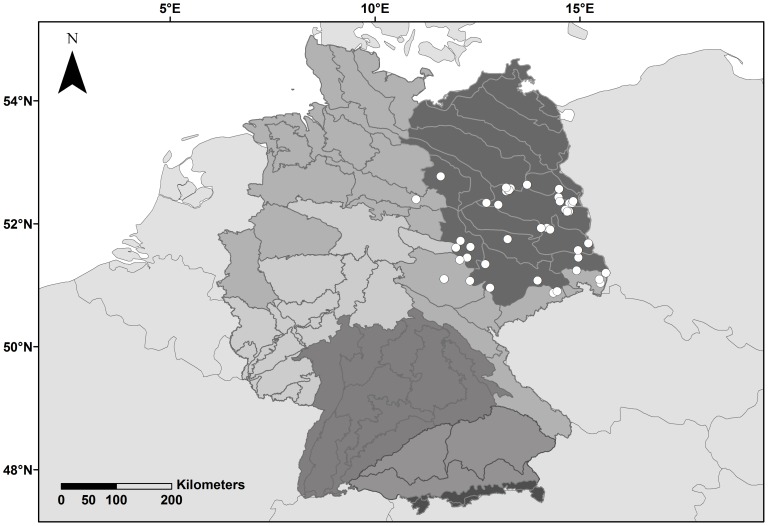
Sampling locations of *Nyctalus noctula* carcasses (open circles) at wind turbines in the Eastern part of Germany. The seven major terrestrial natural regions in Germany [26] are shown in different shades of grey. Macrochores [26] within the major natural regions are indicated by solid lines.

We analyzed fur samples of 136 *N. noctula* found dead below wind energy facilities in the eastern part of Germany between 2002 and 2012. This study benefited from carcass collections at wind turbines that were deposited at federal agencies (e.g. Staatliche Vogelschutzwarte Buckow) or museums (e.g. Central Repository for Natural Science Collections, Univ. Halle-Wittenberg). Legal permits were not required for collecting minute samples of fur from these museum specimens because carcasses were not removed from collections. Bat carcasses were originally collected during fatality searches conducted from July to September of the corresponding year and originated from 45 sites located mostly in the natural region of lowland Northeast Germany [26] (Fig. 1). From each deposited individual, we collected a small fur sample from the dorsal side of the pelage, recorded the sex of the bat and, whenever possible, estimated age according to the closure of the epiphyseal gaps. Additionally, the latitude and longitude of the respective sampling locations were retrieved.

All fur samples were rinsed with 2∶1 chloroform-methanol solution to wash off surface oils and contaminants. Then, samples were dried in a drying oven over 24 hours at 50°C. A subsample of 1 mg (±0.1 mg) was weighed in a silver foil capsule (IVA Analysetechnik e.K. Meerbusch, Germany). All laboratory procedures were previously described in [21] and [19]. Briefly, fur samples were placed in the autosampler (Zero Blank autosampler; Costech Analytical Technologies Inc., Italy) of the elemental analyzer (HT Elementaranalysator HEKAtech GmbH, Wegberg, Germany). Before combustion, samples were flushed in the autosampler for at least 1 hour with chemically pure helium (Linde, Leuna, Germany). We used a Delta V Advantage isotope ratio mass spectrometer (ThermoFischer Scientific, Bremen, Germany) that was connected via an interface (Finnigan Conflo III, ThermoFisher Scientific, Bremen, Germany) with the elemental analyzer. Using laboratory keratin standards with known stable isotope ratios of the non-exchangeable portion of hydrogen, we were able to report only stable isotope ratios of the non-exchangeable hydrogen in fur keratin (δ^2^H_f_; see details in [18], [21]). Analytical precision based on the repeated analyses of laboratory keratin standards was always better than 1 ‰ (one standard deviation of mean ratios). Details on sex (males, females), age, δ^2^H_f_ (‰) and sampling location (ID) of the bat carcasses can be found in Table S1.

### Identification of migrant *N. noctula* via stable hydrogen isotope ratios of fur keratin

We identified migratory individuals among the fatalities following the approach outlined in [13]. For this, we used a reduced major axis regression model (RMA) that correlates δ^2^H_f_ of sedentary bats with mapped values of δ^2^H in mean annual precipitation (δ^2^H_p_; [22]). We used the regression parameters from [13], since this model incorporates, among other sedentary bats, data from sedentary *N. noctula*. Detailed information on the calibration procedures and specifications of the RMA are given in [13]. Based on the regression relationship and the associated uncertainty of the RMA for *N. noctula* [13], individuals were identified as either sedentary or migratory if individual δ^2^H_f_ values were inside or outside, respectively, of the 95% confidence interval of the expected δ^2^H_f_ values for the specific site.

### Influence of spatial, temporal and biological co-variables on δ^2^H_f_

We fitted a linear mixed-effects model exploring the influence of the explanatory variables ‘season’, ‘sex’, ‘migratory behaviour’, ‘latitude’, ‘longitude’, and the two-way interaction of ‘latitude and longitude’ on the dependent variable δ^2^H_f_. We used ‘day of the year’, calculated from the respective date each bat carcass was found, as a proxy for season. The co-variable ‘migratory behaviour’ referred to the categories ‘migratory’ and ‘sedentary’ as calculated from the established RMA (see above). Testing for collinearity between the predictor variables was performed by calculating Pearson’s product moment correlation coefficient *r* as suggested by [27]. ‘Sampling location’ and ‘macrochore’ as defined by [26] were considered for inclusion in the model as a random effect, respectively. Sampling location referred to the actual wind power facility site (n = 45), whereas ‘macrochore’ defined the wider natural regions (n = 12). The sample size ranged from 1 to 16 bats per facility with a mean of 3 individuals per facility. To assess the appropriate structure of the model, we followed a top-down strategy and focused on the optimal structure of the random component first [28]. We applied an analysis of variance (ANOVA) to compare a fixed-effects model fitted by generalized least squares (GLS) with two mixed-effects models, which included ‘sampling location’ or ‘macrochore’ as random effect, respectively (package nmle, [29]). We used restricted maximum likelihood (REML) estimation to first establish the correct random effect structure. Before entering fixed-effect co-variables, we used generalized additive models with three knots (GAM; package mgcv, [30]) to visually check the linearity assumption of the co-variables. The co-variables ‘sex’ and ‘migratory behaviour’ were entered as categorical variables. The identification of the optimal structure of the fixed component was performed following [28]: We fitted the full model with the previously identified optimal random structure using ML estimation and used the likelihood ratio test to identify non-significant terms. Estimates of the final model were then fitted using REML. We tested for homogeneity in residuals using the Kolmogorov-Smirnov normality test with Lilliefors correction (package nortest). All statistical analyses were performed with R 3.0.1 [31] and level of significance was set at 0.05 for all analyses.

### Modeling the geographical origin of migratory bats based on δ^2^H_f_

To delineate the origins of migratory *N. noctula* killed by wind turbines in the δ^2^H_p_ isoscape of Europe, we used an extrapolation approach, i.e. we established a relationship between stable isotope ratios in non-exchangeable hydrogen of bat fur and mean annual precipitation in Europe, accounting for potential geospatial assignment errors. This leads to robust conclusions about the origin of migrant bats (cf. [13], [18], [19]). We used the regression parameters of the RMA for sedentary bats, including sedentary *N. noctula* as published in [13] to convert δ^2^H_f_ values into δ^2^H_p_ values of migratory bats. For each bat fatality, we simulated 10,000 possible δ^2^H_p_ values of the measured δ^2^H_f_, incorporating the estimated variance of local δ^2^H_p_ values based on the RMA. Subsequently, a final value was drawn from a normal distribution with the mean corresponding to the regression value and the standard deviation randomly generated from the gamma distribution. We then created standardized probability density functions (PDFs; package MASS, [32]) ranging between 0 and 1. The PDFs were used to reclassify the δ^2^H_p_ map into isoscape origin probability maps (package raster, [33]) for each sampling location (Isoscape Origin Probability Map *PM_Iso_*). Subsequently, we weighed the resulting probability maps according to the sample size of the respective sampling location and merged them into one final *PM_Iso_* (package raster). Since sex-specific differences in the distribution of vespertilionid bat species have already been documented in North America [34], [35], [36] we applied this spatial extrapolation approach for data of males and females separately.

In order to further refine the projections of our bat origin model, we also included information on species-specific preferences on cardinal headings during spring migration derived from banding data [8]. Recapture data of banded bats link the place of origin and the place of hibernation in the most direct way and we assume that bats return to their breeding area directly. Including information on species-specific cardinal headings during migration as an additional parameter thus helps to narrow the geographical areas of potential origin further down when spatially extrapolating the model. The data processing and statistical treatments applied to the banding data prior to inclusion in the model were previously described in [13]. Briefly, a point-inversion was used to convert the observed flight directions of banded bats during autumn migration into assumed flight directions during spring migration. Subsequently a standardized PDF (package circular; [37]) was created from the flight directions. We then computed the bearing from the single sampling locations for each raster cell of the δ^2^H_p_ grid (package geosphere; [38]) and reclassified the bearing with the flight direction probability (Angular Probability Map *PM_Ang_*). The resulting probability maps were weighed according to sample size per sampling location and then merged into one final *PM_Ang_* (package raster).

We then calculated the refined probability of breeding origin *PM_Breed_* as
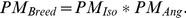
(1)


Subsequently we standardized the *PM_Iso_* map and the refined *PM_Breed_* map between minimum and maximum values. ArcGIS v. 9.3.1 was used to visualize the reclassified raster surface with a resolution of 10′×10′ (arc minutes).

## Results

### Isotopic separation of local and migrant *N. noctula* killed by wind turbines

According to the stable isotope ratio in the non-exchangeable hydrogen of fur keratin, we categorized 28% of individuals as migratory and 72% as local. The proportion of males and females was balanced in local individuals, whereas 62% of bats were females in migrant individuals (Table 1). A considerable proportion of juveniles was recorded among local (38%) as well as among migratory (32%) bats (Table 1).

**Table 1:** Number, sex (males, females), age and δ^2^H_f_ (‰; mean ± SD) of migrant and sedentary *Nyctalus noctula* killed by wind turbines in eastern Germany.

	Migratory			Sedentary		
	Total (m/f)	δ^2^H_f_		Total (m/f)	δ^2^H_f_	
		males	females		males	females
	**37** (14/23)	**−111.7**±6.8	**−122.5**±14.0	**99** (47/52)	**−92.6**±6.3	**−94.1**±7.0
**Adult**	**21** (6/15)	**−115.4**±7.5	**−125.4**±15.1	**25** (9/16)	**−91.7**±5.1	**−98.2**±6.0
**Juvenile**	**12** (6/6)	**−108.9**±5.9	**−118.9**±11.1	**38** (18/20)	**−96.3**±5.9	**−95.4**±6.2
**NA Age**	**4** (2/2)	–	–	**36** (20/16)	**−89.7**±5.7	**−88.4**±5.1

NA = not available.

### Influence of spatial, temporal and biological co-variables on δ^2^H_f_

We did not detect any collinearity in the predictor variables (|*r|* <0.75). All variables indicated a linear relationship (GAM, see Figure S1). The model containing ‘sampling location’ as random intercept performed best, as indicated by minimization of AIC. Information on the full model is given in Table S2. The explanatory variables of the best model in explaining δ^2^H_f_ values contained ‘season’, ‘sex’ and ‘migratory behaviour’ (Table 2), with δ^2^H_f_ values increasing from July to September (‘season’). Local bats and males had significantly higher δ^2^H_f_ values (Table 2). Residuals of the model were normally distributed (Lillefors D = 0.0445, *P* = 0.21).

**Table 2:** Results of the linear mixed-effects model fit by REML for predicting δ^2^H_f_ from ‘season’, ‘sex’ and ‘migratory behaviour’ with ‘sampling location’ as random effect.

Model parameter	Estimate	SE	t-value	*P*
(Intercept)	**−**126.8	9.84	**−**12.9	<0.001
Season	0.1	0.04	3.1	<0.003
Sex (Males)	3.1	1.30	2.4	<0.020
Migratory behaviour (Migrants)	**−**21.0	1.57	**−**13.4	<0.001

Degrees of freedom: 88; number of observations: 136; number of groups (random effect ‘sampling location’): 45. AIC = 947.5, BIC = 964.8, logLik **−**467.8; random intercept (mean 0, SD 6.36), and residual term (mean 0, SD 6.31) were normally distributed.

### Modeling the geographical origin of migratory bats based on δ^2^H_f_

**Figure 2:**
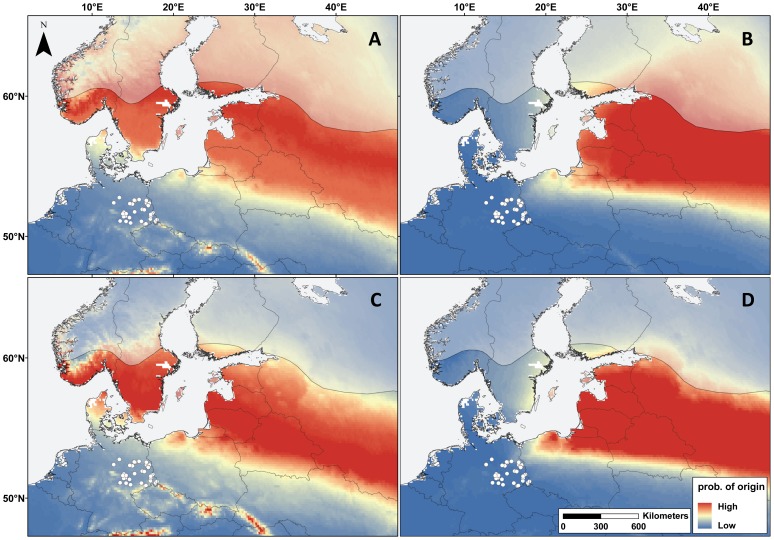
Predicted geographical provenance of the 28% of migratory *Nyctalus noctula (n = 37)*, separated by females (A,B) and males (C,D) found dead below wind turbines in Eastern Germany. Geographical areas marked red are areas of likely breeding origin and those marked blue areas of unlikely breeding origin. Predictions shown in map A and C are based on δ^2^H_f_. Predictions shown in map B and D incorporate additional information on the preferred cardinal heading of *N. noctula* during spring migration. Areas falling outside the distribution range of *N. noctula* according to the IUCN are overlaid with a semi-transparent layer.

A spatial model that was solely based on δ^2^H_f_ documented that migratory *N. noctula* killed by wind turbines in eastern Germany originated from distant populations in the Northern and Northeastern parts of Europe. The projected geographical origins of females included more distant regions than those of males (Fig. 2 A, C). Our second model, further refining the extrapolation of breeding origins by additionally accounting for preferred flight directions of *N. noctula* suggested that male and female individuals most probably originated from Baltic countries (Lithuania, Latvia, Estonia), Belarus and Russia, with the geographical range projected for the origin of female bats extending further to the North and Northeast than for males (Fig. 2 B, D).

## Discussion

In light of a continuously growing wind energy sector in Germany [10], [11] and evidence that large numbers of protected European bats are killed annually at wind turbines [5], [6], an improved understanding of the location of affected source populations is of prime interest and importance for conservation planning. In Europe, Germany is the country with the highest number of established wind power facilities, and recent studies showed that almost 70% of recorded bat fatalities in this country involve species with long distance migratory behaviour (Dürr 2009 cited in [5]). Despite the urgent need to better understand the geospatial origin of bat fatalities, there is only a single pilot study on the catchment area of wind power facilities. This study demonstrates that many non-resident individuals from distant origin are among the recorded fatalities at wind power stations, yet sample sizes were limited in this pilot study [19]. In our current study, we investigated in more detail the spatial origin of noctule bats that were killed at wind turbines in eastern Germany to shed light on the geographical range at which bat populations might be affected by the increased mortality risk at wind turbines. For this, we used a larger data set for noctule bats (*Nyctalus noctula*), which is the European species most often killed by wind turbines in Central Europe (Dürr cited in [5]). Samples originated from a large number of wind turbines in the eastern parts of Germany. We found that the majority of individuals killed at wind turbines in eastern Germany were of local origin, i.e. they came from populations located within the same δ^2^H_p_ isocline as the sites where they were found dead. However, more than a quarter of individuals were identified as long-distance migrants, demonstrating that bat fatalities at wind energy facilities are not only a threat for populations nearby but also for populations at relatively large geographical distance. It is widely assumed that reproduction rates of species are lower at the border of their distribution range than in the core area when suboptimal habitat structures in the periphery of the geographical distribution range limit reproduction. Our finding that a relatively large proportion of bats may have originated from the northern border of the species’ geographical distribution range is thus troubling, considering that birth rates and recruitment may be lower in suboptimal northern habitats than in more suitable habitats of Central Europe. Therefore, individual losses caused by wind turbine-related fatalities may be more significant for northern populations than for populations in Central Europe.

We found a relative high proportion of juveniles among local bats. This finding suggests that effective mitigation measures for operating wind power facilities in the vicinity of roosts should be implemented to substantially reduce fatalities among sedentary breeding populations of noctule bats. Adopting appropriate thresholds for minimum distances between planned wind turbine sites and potential roosts or maternal colonies could constitute an additional powerful management tool. The widespread practice in Germany of keeping a 1-km distance between wind turbine sites and known roosts, however, may prove inefficient in substantially reducing the number of fatalities for juvenile noctule bats because of the large home range of this bat species. Fatalities of juvenile noctule bats were also recorded among migrant individuals, albeit to a lesser extent. With respect to migratory *N. noctula*, our data indicated that adult females might be particularly susceptible to wind turbines. This finding is in contrast to those of studies from North America where more males than females were killed at wind turbines [39], yet this discrepancy may be explained by species-specific and sex-specific foraging or migratory behaviours in European and North American species. Several studies on population dynamics of bat species suggest that adult survival may be more important for population growth than juvenile survival (e.g. [40], [41]). Considering the high philopatry of female noctule bats on the one hand [14], [42] and the low reproduction rate of female bats on the other hand [15], one may infer that recruitment for and stability of noctule bat populations are particularly sensitive to increased mortalities of adult female individuals. Thus, a larger fatality rate of adult female bats at wind turbines may exacerbate negative population effects.

### Influence of temporal and biological factors on measured δ^2^H_f_

We predicted that information on migratory status and seasonality would explain most of the variance in measured δ^2^H_f_ values of noctule bat fatalities. The results of our mixed-effects model, which included ‘sampling location’ as random intercept and consequently incorporated the variance associated with differences between sampling sites, confirmed our prediction in that both explanatory variables had a significant effect on δ^2^H_f_ (Table 2). Migratory bats exhibited substantially lower δ^2^H_f_ values compared with those of local individuals indicating that they originated from geographical regions north of the study area [21], [22]. This is consistent with our hypothesis that killed migrants originated from north and not from south of the site where they were found dead. δ^2^H_f_ values increased with proceeding season, potentially reflecting a decreasing proportion of long-distance migrants from July to September. With regard to the spacious distribution of the sampling locations, which are dispersed over twelve macrochores [26] and which include sites located in lowland as well as mountainous areas, it is not surprising that incorporation of ‘sampling location’ as random factor improved the model fit substantially. Furthermore information on sex contributed to the explanatory power of our model (Table 2), i.e. the model indicated that female *N. noctula* exhibited lower values of δ^2^H_f_ than males, indicating that the summer habitats of females are further to the North and Northeast than those of male noctule bats.

### Isoscape origin models

Our isoscape origin models confirmed the result of the LMM depicting that female migratory *N. noctula* originated from populations at greater geographical distance and dispersed further to the North and Northeast than males (Fig. 2). The spatial model that was solely based on δ^2^H_f_ suggests that breeding origins of noctule bats found in eastern Germany range from Scandinavia and the eastern Baltic countries to Belarus and Russia, which is consistent with findings of our pilot study [19]. Our refined model additionally accounts for preferred flight directions of *N. noctula* during spring migration and, therefore provides more accurate estimation of potential breeding provenance by excluding Scandinavia as a source habitat (Fig. 2). This result is consistent with previous genetic studies in *N. noctula* which document a separation of northern populations from Central and East European populations [14]. Also, banding data established over the past four decades [8], [9], and an advanced isoscape origin model [13] suggest that migratory noctule bats migrating through or hibernating in eastern Germany originate from Poland, Baltic countries or Belarus.

### Assumptions and potential biases

We obtained carcasses during the July to September period of several subsequent years, thus covering the period in which most bat fatalities at wind turbines have been recorded in Germany [6]. However, since we lack profound information on the sampling effort conducted in each respective year, we were not able to account for a potential bias introduced by uneven sampling effort between and within years.

Our isoscape origin model was based on measured stable hydrogen isotope ratios in fur keratin, on a reduced major axis regression established for pre-migratory *N. noctula* as well as sedentary species [13], and on expected flight directions of *N. noctula* during autumn migration. Any violation of one or several of the assumptions underlying our approach could affect the accuracy and precision of our spatial model. For a detailed discussion on uncertainties associated with the use of RMA in *N. noctula* and assumed preferred flight directions obtained from banding data please see the discussion in [13]. In contrast to our previous approach [13], we did not include estimated maximum travel distances of *N. noctula* in our isoscape origin model, since bats were killed en route during autumn migration. Thus, travel distances obtained from noctule bats banded in the breeding habitat and recovered at the hibernacula site are not representative for our data set. While we are convinced that our geographical predictions for individuals identified as long-distance migrants are realistic [13], we do recognize shortcomings in the identification of regional migrants originating from breeding habitats located within the same isocline as our sampling locations, e.g. noctule bats from Poland. These individuals have similar stable isotope ratios of non-exchangeable hydrogen in fur keratin as noctule bats from populations in eastern Germany. Therefore, we subsumed noctule bats from German and Polish populations (except for the northeast of Poland) as local bats. Considering that noctule bats from Poland may as well be long-distance migrants that were killed en route in Germany, the documented proportion of 28% of migratory *N. noctula* have to be considered as a conservative estimate of the proportion of migratory individuals killed by German wind power facilities.

### Conclusions

Here we present the first comprehensive study on the breeding provenance of endangered and protected noctule bats killed at German wind turbines, showing that individuals from local and distant populations are among the recorded fatalities. Our results provide a first step on the way towards an evaluation on how bat fatalities at wind turbines might impact local and regional populations in migratory bat species. Bat populations are difficult to monitor and we are still far from understanding population sizes, demographic parameters, and consequently also potential impacts of fatalities on the viability of bat populations [39]. Yet, the large numbers of bat fatalities at wind turbines each year call for immediate action and an appropriate research agenda on the national and international scale. Our results highlight the importance of implementing effective mitigation measures and developing species and scale-specific conservation approaches on international levels. The efficacy of potential compensatory measures in the proximity of wind turbine facilities on the other hand, e.g. establishing artificial bat boxes [13] or turning intensively used forest stands into ecologically valuable old forest growth, may be helpful for local bat populations but not necessarily for populations of migratory individuals, considering the trans-boundary origin of bats killed by wind power facilities in Europe.

## Supporting Information

Figure S1 **Results of the generalized additive model with three knots to visually check the linearity assumption of the variables used in the ‘full’ linear mixed-effects model.**
(TIF)

Table S1 **Raw data on** ***Nyctalus noctula*** **(n = 136) killed by wind turbines in eastern Germany, identifying sex (males, females), age, δ^2^H_f_ (‰) and sampling location (ID).**
(DOCX)

Table S2 **Results of the ‘full’ linear mixed-effects model fit by REML for predicting δ^2^H_f_ from ‘season’, ‘sex’, ‘migratory behaviour’, ‘latitude’, ‘longitude’ and their two- way interaction ‘latitude:longitude’ with ‘sampling location’ as random factor.**
(DOCX)
