# Supplementary figures and images for: Wind Farm Facilities in Germany Kill Noctule Bats from Near and Far
Source: PLoS One. 2014 Aug 13;9(8):e103106. doi: 10.1371/journal.pone.0103106 (PMC4138012; doi:10.1371/journal.pone.0103106)

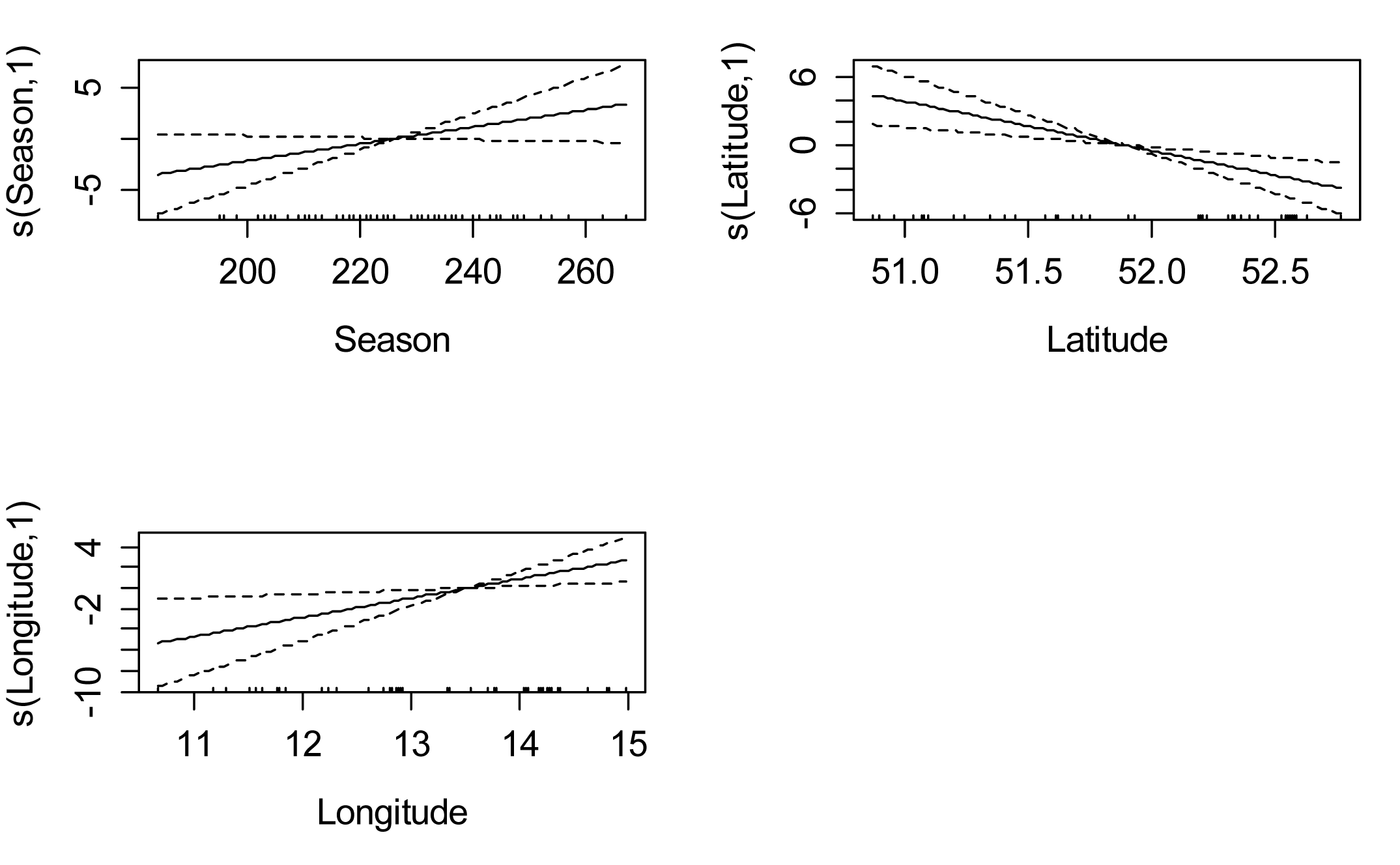

Supplement: Figure S1 — Results of the generalized additive model with three knots to visually check the linearity assumption of the variables used in the ‘full’ linear mixed-effects model. (TIF) [file pone.0103106.s001.tif]
