# Supplementary material for: Wind Farm Facilities in Germany Kill Noctule Bats from Near and Far
Source: PLoS One. 2014 Aug 13;9(8):e103106. doi: 10.1371/journal.pone.0103106 (PMC4138012; doi:10.1371/journal.pone.0103106)
Supplement: Table S1 — Raw data on Nyctalus noctula (n = 136) killed by wind turbines in eastern Germany, identifying sex (males, females), age, δ2Hf (‰) and sampling location (ID). (DOCX) [file pone.0103106.s002.docx]

| **ID** | **Age** | **Sex** | **δ^2^H_f_ (‰)** | **Sampling Location** |
| --- | --- | --- | --- | --- |
| 1 | NA | F | -86.60 | 6 |
| 2 | NA | M | -89.22 | 6 |
| 3 | NA | M | -83.75 | 6 |
| 4 | NA | M | -94.84 | 1 |
| 5 | NA | F | -86.29 | 6 |
| 6 | NA | M | -79.89 | 2 |
| 7 | NA | F | -88.66 | 13 |
| 8 | NA | F | -86.26 | 13 |
| 9 | NA | M | -97.62 | 10 |
| 10 | NA | M | -89.34 | 13 |
| 11 | NA | M | -99.03 | 10 |
| 12 | NA | F | -78.91 | 10 |
| 13 | NA | M | -83.14 | 13 |
| 14 | NA | F | -89.20 | 13 |
| 15 | NA | F | -85.98 | 13 |
| 16 | NA | F | -87.32 | 13 |
| 17 | NA | F | -81.42 | 13 |
| 18 | NA | F | -92.85 | 13 |
| 19 | NA | M | -91.00 | 13 |
| 20 | NA | F | -84.28 | 13 |
| 21 | NA | M | -89.21 | 13 |
| 22 | NA | F | -85.89 | 13 |
| 23 | NA | M | -88.10 | 13 |
| 24 | NA | M | -85.51 | 13 |
| 25 | NA | M | -80.77 | 10 |
| 26 | NA | F | -115.99 | 10 |
| 27 | NA | M | -110.04 | 9 |
| 28 | NA | M | -84.70 | 6 |
| 29 | NA | F | -94.20 | 10 |
| 30 | NA | M | -93.21 | 6 |
| 31 | NA | M | -92.12 | 4 |
| 32 | NA | M | -99.16 | 7 |
| 33 | NA | M | -107.41 | 9 |
| 34 | NA | M | -92.81 | 10 |
| 35 | NA | F | -95.19 | 9 |
| 36 | NA | F | -107.27 | 9 |
| 37 | NA | M | -92.79 | 9 |
| 38 | NA | M | -88.20 | 10 |
| 39 | NA | F | -97.10 | 13 |
| 40 | juvenile | M | -102.13 | 27 |
| 41 | adult | M | -87.80 | 36 |
| 42 | adult | M | -86.62 | 44 |
| 43 | adult | F | -100.73 | 36 |
| 44 | juvenile | M | -98.66 | 36 |
| 45 | juvenile | M | -104.56 | 36 |

| **ID** | **Age** | **Sex** | **δ^2^H_f_ (‰)** | **Sampling Location** |
| --- | --- | --- | --- | --- |
| 46 | juvenile | M | -99.17 | 36 |
| 47 | juvenile | F | -82.87 | 36 |
| 48 | juvenile | F | -125.23 | 36 |
| 49 | juvenile | F | -104.41 | 44 |
| 50 | adult | M | -93.35 | 30 |
| 51 | juvenile | M | -85.77 | 36 |
| 52 | juvenile | M | -105.87 | 34 |
| 53 | adult | F | -108.02 | 41 |
| 54 | juvenile | F | -104.62 | 36 |
| 55 | adult | F | -97.97 | 44 |
| 56 | adult | F | -107.02 | 36 |
| 57 | juvenile | F | -89.95 | 44 |
| 58 | adult | F | -120.85 | 27 |
| 59 | juvenile | M | -94.42 | 11 |
| 60 | juvenile | F | -103.57 | 41 |
| 61 | adult | F | -94.84 | 31 |
| 62 | juvenile | F | -96.61 | 44 |
| 63 | juvenile | F | -95.03 | 22 |
| 64 | juvenile | F | -96.79 | 33 |
| 65 | juvenile | M | -105.64 | 28 |
| 66 | juvenile | M | -107.04 | 28 |
| 67 | juvenile | F | -133.69 | 22 |
| 68 | juvenile | M | -100.71 | 24 |
| 69 | juvenile | F | -81.98 | 23 |
| 70 | juvenile | M | -94.08 | 44 |
| 71 | juvenile | M | -87.43 | 27 |
| 72 | juvenile | F | -92.50 | 27 |
| 73 | juvenile | F | -122.81 | 15 |
| 74 | juvenile | M | -89.72 | 18 |
| 75 | adult | F | -102.91 | 18 |
| 76 | adult | F | -123.66 | 40 |
| 77 | adult | F | -92.16 | 18 |
| 78 | juvenile | F | -96.45 | 22 |
| 79 | adult | F | -106.26 | 18 |
| 80 | juvenile | F | -98.04 | 15 |
| 81 | adult | F | -104.55 | 37 |
| 82 | juvenile | M | -103.27 | 20 |
| 83 | juvenile | M | -97.98 | 24 |
| 84 | juvenile | M | -95.70 | 24 |
| 85 | juvenile | F | -96.44 | 24 |
| 86 | juvenile | M | -106.20 | 28 |
| 87 | adult | F | -85.47 | 34 |
| 88 | juvenile | M | -95.27 | 27 |
| 89 | juvenile | M | -87.90 | 44 |
| 90 | juvenile | F | -92.00 | 41 |
| 91 | juvenile | M | -93.41 | 26 |
| 92 | juvenile | F | -96.76 | 41 |
| 93 | juvenile | F | -92.50 | 15 |

| **ID** | **Age** | **Sex** | **δ^2^H_f_ (‰)** | **Sampling Location** |
| --- | --- | --- | --- | --- |
| 94 | adult | M | -81.62 | 22 |
| 95 | juvenile | F | -102.96 | 44 |
| 96 | juvenile | F | -119.61 | 20 |
| 97 | adult | F | -93.94 | 28 |
| 98 | adult | F | -141.55 | 39 |
| 99 | adult | M | -118.00 | 42 |
| 100 | adult | M | -95.97 | 43 |
| 101 | adult | F | -104.55 | 39 |
| 102 | adult | F | -90.70 | 16 |
| 103 | adult | M | -95.20 | 17 |
| 104 | adult | F | -101.82 | 12 |
| 105 | adult | F | -117.93 | 39 |
| 106 | adult | F | -131.40 | 32 |
| 107 | adult | F | -149.52 | 25 |
| 108 | adult | M | -106.74 | 38 |
| 109 | adult | M | -93.42 | 35 |
| 110 | juvenile | F | -106.29 | 17 |
| 111 | juvenile | F | -92.78 | 17 |
| 112 | juvenile | F | -98.75 | 45 |
| 113 | NA | F | -94.58 | 19 |
| 114 | juvenile | F | -105.47 | 17 |
| 115 | juvenile | F | -92.91 | 8 |
| 116 | juvenile | M | -107.31 | 3 |
| 117 | juvenile | M | -96.69 | 8 |
| 118 | adult | M | -126.29 | 14 |
| 119 | adult | F | -107.72 | 21 |
| 120 | juvenile | M | -120.93 | 29 |
| 121 | juvenile | M | -106.14 | 29 |
| 122 | adult | M | -116.79 | 14 |
| 123 | adult | F | -102.34 | 8 |
| 124 | adult | F | -149.52 | 25 |
| 125 | adult | F | -141.55 | 39 |
| 126 | adult | F | -131.40 | 32 |
| 127 | adult | F | -126.06 | 35 |
| 128 | adult | M | -118.00 | 42 |
| 129 | adult | F | -117.93 | 39 |
| 130 | adult | M | -106.74 | 38 |
| 131 | adult | F | -104.55 | 39 |
| 132 | adult | F | -101.82 | 12 |
| 133 | adult | F | -101.78 | 5 |
| 134 | adult | M | -95.97 | 43 |
| 135 | adult | M | -95.20 | 17 |
| 136 | adult | F | -90.70 | 16 |
