# Supplementary material for: Wind Farm Facilities in Germany Kill Noctule Bats from Near and Far
Source: PLoS One. 2014 Aug 13;9(8):e103106. doi: 10.1371/journal.pone.0103106 (PMC4138012; doi:10.1371/journal.pone.0103106)
Supplement: Table S2 — Results of the ‘full’ linear mixed-effects model fit by REML for predicting δ2Hf from ‘season’, ‘sex’, ‘migratory behaviour’, ‘latitude’, ‘longitude’ and their two- way interaction ‘latitude:longitude’ with ‘sampling location’ as random factor. (DOCX) [file pone.0103106.s003.docx]

| **Model parameter** | **Estimate** | **SE** | **df** | **t-value** | ***P*** |
| --- | --- | --- | --- | --- | --- |
| (Intercept) | -77.80 | 1224.66 | 88 | -0.1 | <0.950 |
| Season | 0.14 | 0.04 | 88 | 3.3 | <0.001 |
| Sex (Males) | 2.95 | 1.30 | 88 | 2.3 | <0.026 |
| Migratory behaviour (Migrators) | -20.65 | 1.57 | 88 | -13.2 | <0.001 |
| Latitude | -1.50 | 23.66 | 41 | -0.1 | <0.950 |
| Longitude | 7.92 | 92.34 | 41 | 0.1 | <0.932 |
| Latitude:Longitude | -0.12 | 1.78 | 41 | -0.1 | <0.949 |
| Number of observations: 136; number of groups (random effect ‘sampling location’): 45. AIC=939.4, BIC=965.1, logLik -460.7; random intercept (mean 0, SD 6.0), and residual term (mean 0, SD 6.32) were normally distributed; model residuals were normally distributed (Lillefors D=0.0456, *P*=0.18). | | | | | |
